# The taxonomy of subjective cognitive decline: proposal and first clinical evidence from the Geneva memory clinic cohort

**DOI:** 10.21203/rs.3.rs-2570068/v1

**Published:** 2023-02-13

**Authors:** Federica Ribaldi, Rafael Palomo, Daniele Altomare, Max Scheffler, Frederic Assal, Nicholas J. Ashton, Henrik Zetterberg, Kaj Blennow, Marc Abramowicz, Valentina Garibotto, Christian Chicherio, Giovanni B. Frisoni

**Affiliations:** University of Geneva; University of Geneva; University of Geneva; University of Geneva; University of Geneva; University of Geneva; University of Geneva; University of Geneva; University of Geneva; University of Geneva; University of Geneva; University of Geneva

**Keywords:** Subjective cognitive decline, Alzheimer’s disease, Dementia

## Abstract

**Background:**

Subjective Cognitive Decline (SCD) is characterized by subjective cognitive complaints without objective cognitive impairment and is considered a risk factor for cognitive decline and dementia. However, most SCD patients will not develop neurodegenerative disorders, yet they may suffer from minor psychiatric, neurological, or somatic comorbidities. The aim of the present study is to provide a taxonomy of the heterogeneous SCD entity by isolating homogenous SCD subgroups with specific clinical features and cognitive trajectories.

**Methods:**

Participants were fifty-five SCD individuals consecutively recruited at the Geneva Memory Center. Based on clinical reports, they were classified into three clinically pre-defined subgroups: (i) those with psychological or psychiatric comorbidities (Psy), (ii) those with somatic comorbidities (SomCom), (iii) and those with no apparent cause (NAC). Baseline demographics, clinical, cognitive, and biomarker differences among the SCD subgroups were assessed. Longitudinal cognitive changes (average 3 years follow-up) were modeled using a linear mixed model.

**Results:**

Out of the 55 SCD cases, 16 were SomCom, 18 Psy, and 21 NAC. 47% were female, mean age was 71 years. We observed higher frequency of *APOE* ε4 carriers in NAC (53%) compared to SomCom (14%) and Psy (0%, P=0.023) and lower level of plasma Aβ_42_ in NAC (6.8±1.0) compared to SomCom (8.4±1.1; P=0.031). SomCom subjects were older (74 years) than Psy (67 years, P=0.011), and had greater medial temporal lobe atrophy(1.0±1.0) than Psy (0.2±0.6) and NAC (0.4±0.5, P=0.005). SomCom have worse episodic memory performances(14.5±3.5) than Psy (15.8±0.4) and SomCom (15.1±0.7, P=0.032). We observed a slightly steeper, yet not statistically significant, cognitive decline in NAC (β=−0.48) compared to Psy (β=−0.28) and SomCom (β=−0.24).

**Conclusions:**

NAC feature higher proportion of *APOE* ε4 carriers, lower plasma Aβ42, worse memory performance, and a trend towards steeper cognitive decline than SomCom and Psy. Taken together, these findings suggest that NAC are at higher risk of cognitive decline due to AD. The proposed clinical taxonomy might be implemented in clinical practice to identify SCD at higher risk. However, such taxonomy should be tested on an independent cohort with larger sample size.

## Introduction

Subjective cognitive decline (SCD) can be defined as the perception of persistent decline in cognitive functions associated with normal performance on standardized cognitive tests.[1, 2]

The concept of SCD was formalized in 2014 by Jessen and colleagues, and has been updated in 2020 by the same group.[1, 2] The authors described the criteria for SCD as a pre-Mild Cognitive Impairment (MCI) condition, consisting of self-experienced persistent cognitive decline in comparison with a previously normal status and normal cognitive performance (adjusted by age, gender and education). Jessen and colleagues proposed features associated with an increased risk of cognitive decline such as the subjective decline in memory irrespective of other domains, the onset of SCD within the past 5 years, onset at 60 years and older, the concern associated with SCD, seeking of medical help, persistence over time and confirmation by an observer. However, these features are common among cognitively unimpaired older adults, and the authors have not proposed a clinical taxonomy to distinguish progressors vs non-progressors SCD.

The prevalence of SCD in the general population is 11%, [3] while in memory clinics is between 20% and 35%. [4, 5] It has been observed that the SCD population has a higher prevalence of abnormal Alzheimer’s Disease (AD) biomarkers, [6] such as amyloid deposition and medial temporal lobe atrophy, than the general population without cognitive complaints. [7–10] Hence, this condition is associated with a higher risk of developing cognitive impairment and dementia, [6] yet most SCD do not develop cognitive deficits. [2] Indeed, Slot and colleagues showed that the incident rate of dementia for 1000 persons-years is around 20% in memory clinics patients and 15% in community-dwelling SCD individuals. [5]

For the previously cited reasons, it is important to identify features associated with an early cognitive decline in the SCD population. In particular, amyloid and tau biomarkers are well known to increase the risk of developing AD and can be assessed through PET (expensive technique implying exposure to ionising radiation), CSF (regarded as an invasive technique with need of training), or blood tests (not yet validated and implemented in clinical practice). [11, 12] Consequently, the assessment of AD biomarkers in all cognitively unimpaired individuals consulted in memory clinics is currently not be feasible. Thus, it is relevant to identify easy-to-collect clinical features associated with cognitive complaints and defining individuals with greater risk of cognitive decline in the SCD population. Furthermore, there is a need to develop practical guidelines for physicians to assess these variables and use them in their daily practice to identify the at-risk SCD individuals. [2, 13] Among them, affective symptoms, personality traits, multimorbidity, and poor health are the most studied variables so far. [14–18]

The present study aims to provide a taxonomy of the heterogeneous clinical SCD entity by isolating homogeneous SCD subgroups with specific clinical and cognitive features and evaluate the associated cognitive trajectories.

## Methods

### 2.1. Population

For the present study, we included 55 SCD patients consecutively recruited at the Geneva Memory Center with available clinical and neuropsychological data. The individuals were classified as SCD if they reported cognitive complaints to the physician, without objective evidence of impairment. [19] The diagnostic workup includes clinical, neurological, and neuropsychological assessments as well as an MRI scan. Thanks to several interconnected ongoing research studies, some patients also undergo amyloid, tau PET scans, APOE genotyping, and blood-based biomarkers assessment. [19]

The neuropsychological battery assessed global cognition (Mini-Mental State Examination, MMSE), memory (3 objects 3 places; Free and Cued Selective Reminding Test delayed recall, FCSRT; digit span), language (category and phonemic fluency), attention (Trial Making Test, TMT, A, digit symbol forward), executive functions (TMT B, TMT B-A, digit symbol backward), and visuospatial abilities (Clock). Anxiety and depression were also assessed (Hospital Anxiety and Depression Scale, HADS).

49 out of 55 subjects underwent neuropsychological assessment at follow-up (mean ± SD follow-up time: 3.1 ± 1.2 years). 32 of them underwent in-person MMSE, and 17 of them telephone MMSE. It has been proven that the telephone version of the MMSE is strongly correlated with the clinical version. [20] Therefore, we converted the telephone MMSE scores into the in-person MMSE scores using the conversion table provided by Newkirk et al. 2004. [20]

### 2.2. Neuroimaging biomarkers

Structural MRI 3D T1-weighted images were acquired using a Magnetom Skyra 3T imager (Siemens Healthineers, Erlanger, Germany). Left and right hippocampal volumes were extracted using automated segmentation from FreeSurfer version 7.0 (recon-all - https://surfer.nmr.mgh.harvard.edu/), and then averaged and adjusted by total intracranial volume. Medial temporal lobe atrophy scale (MTA) and age-related white matter changes scale (ARWMC) were assessed by expert neuroradiologists (MS).

Amyloid-PET images were acquired using 18F-Florbetapir (50 minutes after injection of 200 MBq, 3 5-minute frames) or 18F-Flutemetamol (90 minutes after injection of 150 MBq, 4 5-minutes image frames) tracers. Tau-PET images were acquired using 18F-Flortaucipir (75 minutes after injection of 180 MBq, 6 5-minutes frames).

All PET images were reconstructed using a 3D OSEM iterative reconstruction with 4–6 iterations, 5–8 subsets and applying a 2mm Gaussian filter at Full Width and Half Maximum (FWHM), corrected for randoms, dead time, normalization, scatter, attenuation, and sensitivity.

PET images were processed using an in-house pipeline based on SPM12 (Wellcome Department of Cognitive Neurology, London, UK) as described in Dodich et al. 2020 [21] and standardized uptake value ratio (SUVr) was extracted using the AAL3 atlas. Amyloid-PET SUVr was then converted into Centiloid scale according to the GAAIN guidelines. Global tau-PET SUVr was computed as average across amygdala, parahippocampal gyrus, mid-occipital and inferior temporal cortices. [21]

Amyloid positivity and tau positivity was evaluated by an expert nuclear medicine physician (VG).

### 2.3. Blood-based biomarkers

Plasma samples were collected in EDTA tubes at the Geneva Memory Center, kept for 2 hours at room temperature before centrifugation (1700g 15min), aliquoted as 500uL in 1.2mL polypropylene tubes and stored at −80°C in the local biobank until the time of shipment. Aliquots were shipped under protected conditions and analyzed at the Clinical Neurochemistry Laboratory, University of Gothenburg, Sweden. Plasma Aβ_42_, Aβ_40_, and NfL concentrations were measured using commercially available Single molecule array (Simoa) assays on an HD-X Analyzer according to instructions from the kit manufacturer (Quanterix, Billerica, MA), while p-tau181 concentration was measured using an in-house Simoa methods developed at the Clinical Neurochemistry Laboratory, University of Gothenburg, Sweden.[22]

Biomarkers concentrations were measured by board-certified laboratory technicians who were blinded to clinical data.

### 2.4. SCD subgroups classification

The criteria for SCD subgroups were defined a priori by a neuropsychologist (CC) and a neurologist (GBF) based on clinical expertise and observations of SCD patients consulted at the Geneva Memory Center in the past 5 years. The SCD subgroups were the following: psychiatric (Psy), somatic comorbidity (SomCom), and no apparent cause (NAC). Criteria for classification are described in Table 1. Briefly, the Psy profile refers to patients having psychological and psychiatric comorbidities potentially explaining their cognitive complaints (Table 1). The SomCom refers to patients having multiple pathologies with a high impact on their lifestyle and/or augmenting the risk of dementia. The NAC subgroup refers to patients without any cause potentially explaining their SCD.

Anonymized medical reports from the first consultation at the Geneva Memory Center were used for patients’ classification into one of the three SCD subgroups depending on their profile by a trained physician (RP). RP was blinded to clinical and biomarker results.

### 2.5. Statistical analyses

Continuous variables are reported as mean and standard deviation, and categorical variables as number and percentage. Baseline demographics, clinical, cognitive and biomarkers differences among SCD subgroups were assessed using a Kruskal-Wallis rank sum test for continuous variables and a proportion test for categorical variables. To investigate the effect of the SCD subgroup on cognitive changes over time, linear mixed effect models were performed with MMSE as the dependent variables, and SCD subgroup, time (years), and SCD subgroup*time interaction as the independent variables. The models were adjusted for age and education. Random intercept and random slope were considered to account for individual differences at baseline as well as for individual change over time.

All the analyses were performed using R 4.0.5 (http://www.r-project.org).

## Results

Out of 55 subjects, 26 were female (48%), mean age was 69 years. 16 patients were classified in the SomCom subgroup, 18 in the Psy subgroup and 21 in the NAC subgroup. Table 2 shows the demographics, clinical, cognitive and biomarkers differences among the three SCD subgroups. Individuals in the SomCom subgroup were older than the Psy (74 ± 5 vs 67 ± 7, P = 0.011). The prevalence of females was similar among the three subgroups (44% in SomCom, 50% in Psy, and 48% in NAC, P = 0.935). We observed no statistically significant differences in education (15.4 ± 3.9 in SomCom, 16.0 ± 4.5 in Psy, and 16.5 ± 3.7 in NAC; P = 0.547).

The Psy subgroup had higher levels of anxiety (9.2 ± 3.5) compared with both SomCom (5.6 ± 3.4) and NAC (6.8 ± 2.8, P = 0.009) subgroups.

There is a higher prevalence of APOE e4 carriers in the NAC subgroup (53%, 8/15) compared to SomCom (14%, 1/7) and Psy (0%, 0/7, P = 0.023). MTA scale was significantly higher in SomCom (1.0 ± 1.0) than in NAC (0.5 ± 0.5) and Psy (0.24 ± 0.59), and in NAC than in Psy (P = 0.005). Levels of plasma Aβ42 are significantly lower in NAC (6.78 ± 1.04) than in SomCom (8.38 ± 1.08, P = 0.031). However, amyloid-PET SUVr levels were not significantly different among subgroups.

The three subgroups had similar levels of global cognition (MMSE, 28.5 ± 1.5 in SomCom, 29.1 ± 0.8 in Psy, and 28.7 ± 0.8 in NAC, p = 0.317). However, we observed worse episodic memory performance in NAC (46.3 ± 2.0) and SomCom (45.6 ± 2.4) than in Psy (47.4 ± 1.0, P = 0.021) considering the FCSRT Total Immediate Recall test; and in SomCom (14.5 ± 3.5) than in Psy (15.8 ± 0.7) and NAC (15.8 ± 0.7) considering the FCSRT Delayed Recall test. No differences in other cognitive domains were observed.

**Figure 1:**
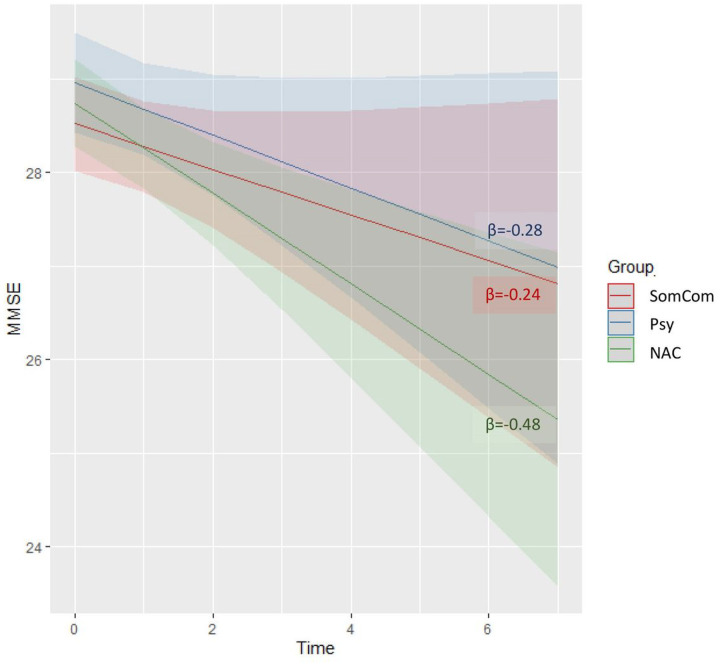
Cognitive trajectories according to SCD subgroup. The X-axis represents time in years. Lines represent predicted mean trajectories obtained from the linear mixed model. Shaded areas indicates 95% confidence intervals. Higher MMSE values indicate better performance. Slopes were not statistically significant (p= 0.268).

As it is shown in Fig. 1, we observed a slightly steeper cognitive decline in the NAC subgroup (N = 20) compared to the Psy (N = 15), and SomCom (N = 14) yet not statistically significant (NAC β=−0.48, SomCom β=−0.24 vs Psy β=−0.28, P = 0.268).

## Discussion

This study aimed to provide a taxonomy of the heterogeneous clinical SCD entity by isolating homogeneous SCD subgroups with specific cognitive features and trajectories, allowing to identify the subgroup with a higher risk of dementia. To this end, 55 SCD individuals were classified into three different SCD subgroups defined a priori based on clinical experience. Our results identified significant differences existed among the SCD subgroups, supporting the body of literature describing the heterogeneity of this population.[13]

Results showed a difference in age among the groups, with older individuals in the SomCom subgroup, followed by the NAC group and finally the Psy one. As the incidence and number of pathologies tend to increase with age, it is not surprising that the SomCom group was older and presented the worst cognitive performance on episodic memory tests. The predictive value of SCD on incipient dementia decreases with age.[23] As a consequence, the cognitive complaints reported by the individuals in the SomCom group could be explained by a global complaint on health status and might be less likely to worsen over time compared to the other groups.

As an internal validation of the proposed taxonomy, we observed higher levels of depression and anxiety in the Psy subgroup. It has been reported in the literature that late-life depression occurring after 65 years old can be either the cause of cognitive impairment or can be a symptom of a coexisting neurodegenerative pathology. [24] Hence the importance of studying the longitudinal fluctuation of depressive symptoms is probably more useful than a mere assessment of their presence in a specific moment. If depressive symptoms begin before cognitive symptoms, it is more likely a psychiatric issue. [24] On the other hand, if cognitive symptoms precede depression signs, there is a higher chance of underlying dementia. [24] Moreover, a recent study reported that subclinical depressive symptoms are not associated with brain amyloidosis in cognitively healthy older adults. [25] This could result in a lower risk of developing Alzheimer’s disease dementia in the Psy subgroup.

Medial temporal lobe atrophy was higher in the SomCom group. This result is consistent with their lower memory performance, and may be explained by the age difference of the subgroups as growing old is associated with increased brain atrophy.[26–29]

Another interesting finding was the higher prevalence of APOE e4 carriers in the NAC group. APOE e4 carriers have a 50% lifetime risk of developing AD if they are homozygotes, and 20–30% if heterozygotes. In comparison, the lifetime risk of developing AD irrespective of APOE genotype is 11% for men and 14% for women. [30, 31] It has been shown that *APOE* ε4 or SCD increases the risk of MCI and dementia (hazard ratio: 1.4–1.8) compared to a population without one of these characteristics. [32] However, their simultaneous presence increases, even more, the association with MCI and dementia (hazard ratio: 2.6). [32] It has been demonstrated that *APOE* ε4 is associated with a higher risk of amyloid pathology. [33] Therefore, it is not surprising that the NAC group, featuring a higher proportion of *APOE* ε4 carriers, has the highest plasma amyloid levels. The concentration of plasma Aβ42 calculated is inversely proportional to the cerebral amyloid load, [34] and there is a growing body of literature investigating the association between plasma Aβ42 and cognitive decline, implying that lower Aβ42 is associated with steeper cognitive decline over time. [35] Therefore, the NAC shows higher prevalence of features associated with a higher risk of developing dementia.

Finally, longitudinal results showed a trend for cognitive decline in the NAC group. The results were not statistically significant probably because of the limited sample size. If the observed difference is in the future confirmed by an additional independent study, it would represent a strong additional argument in favor of the hypothesis that the NAC group is the one most at risk.

There are some limitations to this study. First, we could not test the SCD + features and the comparison between the predictive value of them compared to our taxonomy. Second, we are aware of the relative sample size.

### 5.1. Conclusions

In conclusion, the results suggest that SCD patients with somatic comorbidities have lower but stable memory performance, while those without somatic or psychiatric comorbidities might be those at higher risk of future cognitive impairment and dementia due to Alzheimer’s disease. The proposed taxonomy might be easily implemented in clinical practice by assessing basic clinical information. This taxonomy should be tested on an independent cohort with a bigger sample size.

The present study, if replicated on an independent clinical cohort, could pave the way for an SCD clinical taxonomy, and give practical information to clinicians so they can stratify the risk of their patients depending on their clinical profile based on basic and easy-to-collect information.

## Figures and Tables

**Table 1:** Criteria for SCD subgroups’ classification. Cut-off = 2. Patients are assigned to the Psy or SomCom groups if the pertinent score is 2 or 3. If the Psy score and the SomCom score are equal, the patient is classified as NAC. If the score in both categories is lower than 2, the patient is classified as NAC. If the score is 2 for Psy and 3 for SomCom or vice versa, the highest will drive the classification.

Score					
1		2		3	
Psy	SomCom	Psy	SomCom	Psy	SomCom
Subtle psychiatric disorders, e.g.: - Subtle anxiety - Subtle depression - Subtle personality disorders	Cardiovascular risk factors (CVRF, e.g.): - Hypertension - Metabolic syndrome - Obesity - Diabetes - Family history of stroke or cardiac infarction	Psychiatric disorders, e.g.: - Major depression or >2 - depressive episodes medically treated in the last 10 years - Generalized anxiety disorders ADHD Personality disorders	1 untreated CVRF or >3 CVRF Chronic diseases requiring chronic corticoid or immunosuppressor treatment (e.g.): - HIV - Polymyalgia rheumatic Neurological comorbidity with intermittent neurological dysfunction (e.g.): - Epilepsy - Migraine with aura	Treated severe psychiatric disorders (e.g.): - Schizophrenia - Bipolar disorder	Chronic/Acute neurologic comorbidity associated with permanent motor or sensitivity loss (e.g.): - Lateral amyotrophic sclerosis - Multiple sclerosis - Stroke Physical comorbidity requiring >2 surgical interventions (e.g.): - Cures of inguinal hernia Cancer comorbidity currently treated or not in remission Cardiovascular comorbidity requiring surgical/endovascular intervention > 1 untreated CVRF

**Table 2:** Demographics, clinical, cognitive and biomarkers features of the sample by SCD subgroups.

Variables	SomCom (N = 16)	Psy (N = 18)	NAC (N = 21)	P
**Demographics and clinical features**				
Age	74 ± 5*	67 ± 7*	70 ± 5	**0.011**
Gender, female	7 (44%)	9 (50%)	10 (48%)	0.935
Education	15.4 ± 3.9	16.0 ± 4.5	16.5 ± 3.7	0.547
Depression^a^	3.5 ± 1.9	5.8 ± 3.5	4.3 ± 3.0	0.172
Anxiety^a^	5.6 ± 3.4*	9.2 ± 3.5*-	6.8 ± 2.8-	**0.009**
HADS Total^a^	9.1 ± 4.4*	15.0 ± 5.9*-	11.1 ± 5.0-	**0.014**
Somatic comorbidity	3.8 ± 2.0	2.4 ± 1.5	3.0 ± 1.8	0.111
**Biomarkers**				
APOE e4 carriers^b^	1/7 (14%)*^	0/7 (0%)*-	8/15 (53%)-^	**0.023**
MT^c^	1.0 ± 1.0*^	0.2 ± 0.6*-	0.5 ± 0.5-^	**0.005**
Hippocampal volume^d^	3748 ± 623	3840 ± 641	3755 ± 630	0.647
ARWMC^e^	6.1 ± 6.3	5.8 ± 6.2	5.6 ± 5.7	0.902
Amyloid Centiloid^d^	15 ± 30	10 ± 22	23 ± 39	0.804
Amyloid positivity	4 (25%)	3 (17%)	9 (43%)	0.182
Tau Global SUVr^f^	1.12 ± 0.11	1.18 ± 0.14	1.10 ± 0.17	0.315
Tau positivity^f^	0/12 (0%)	0/9 (0%)	1/10 (10%)	0.338
Plasma ptau181^g^	10.3 ± 6.8	9.3 ± 4.7	8.6 ± 4.4	0.975
Plasma ab40^h^	136 ± 31	123 ± 37	108 ± 23	0.109
Plasma ab42^h^	8.4 ± 1.1^	7.5 ± 2.1	6.8 ± 1.0^	**0.031**
Plasma Gfap^h^	140 ± 41	137 ± 51	143 ± 73	0.973
Plasma Nfl^h^	22.5 ± 9.8	23.4 ± 8.3	17.6 ± 5.2	0.162
**Cognition**				
MMSE^a^	28.5 ± 1.5	29.1 ± 0.8	28.7 ± 0.8	0.317
Clock^a^	9.5 ± 0.6	9.3 ± 1.2	9.5 ± 0.9	0.933
**Demographics and clinical features**				
Three Objects Three Places^i^	8.6 ± 1.2	8.8 ± 0.3	8.9 ± 0.4	0.594
FCSRT Free Immediate recall^a^	28.8 ± 4.8	32.3 ± 5.7	30.1 ± 5.0	0.185
FCSRT Total Immediate recall^a^	45.6 ± 2.4*	47.4 ± 1.0*-	46.3 ± 2.0-	**0.021**
FCSRT Free Delayed recall^a^	11.5 ± 2.4	12.8 ± 1.7	12.6 ± 1.6	0.144
FCSRT Total Delayed recall^a^	14.5 ± 3.5*^	15.8 ± 0.4*	15.8 ± 0.7^	**0.032**
Digit Span^c^	24.5 ± 4.5	26.2 ± 4.1	24.3 ± 4.7	0.396
Category Fluency^l^	17.5 ± 4.1	20.1 ± 5.0	20.7 ± 4.1	0.088
Phonemic Fluency^l^	15.3 ± 6.3	19.7 ± 6.4	18.6 ± 6.4	0.254
TMT A^a^	43.3 ± 13.5	38.8 ± 14.4	46.6 ± 26.7	0.550
TMT B^a^	109 ± 37	88 ± 42	103 ± 49	0.100
TMT B-A^a^	65 ± 34	50 ± 32	56 ± 34	0.202
Digit Symbol^l^	52 ± 12	62 ± 19	53 ± 13	0.278

Abbreviations. HADS=hospital anxiety and depression scale; APOE=Apolipoprotein; MTA=medial temporal lobe atrophy scale; ARWMC=age related white matter changes scale; MMSE=Mini-Mental State Examination; FCSRT=Free and Cued Selective Reminding Test; TMT=Trail Making Test

Missing values: a=1; b=26 (9,11,6); c=3; d=2; e=6; f=24; g=34; h=19; i=8; l=4

Post-hoc comparisons:

* significant differences between SomCom and Psy;

- significant differences between Psy and NAC;

^ significant differences between Som and NAC.

## Data Availability

The data that support the findings of this study are available from the corresponding author (FR), upon reasonable request.
